# ﻿Resurrection of *Drypetesnienkui* (Putranjivaceae), endemic to Hainan, China

**DOI:** 10.3897/phytokeys.206.87737

**Published:** 2022-09-12

**Authors:** Geoffrey A. Levin

**Affiliations:** 1 Illinois Natural History Survey, Prairie Research Institute, University of Illinois, 1816 S Oak St., Champaign, Illinois 61820, USA University of Illinois Champaign United States of America; 2 Research and Collections, Canadian Museum of Nature, P.O. Box 3443, Station D, Ottawa, Ontario K1P 6P4, Canada Research and Collections, Canadian Museum of Nature Ottawa Canada; 3 Naturalis Biodiversity Centre, PO Box 9517, 2300 RA Leiden, Netherlands Naturalis Biodiversity Centre Leiden Netherlands

**Keywords:** Asia, China, *Drypetes*, Hainan, Putranjivaceae

## Abstract

*Drypetesnienkui* (Putranjivaceae), described from Hainan, China, has long been treated as a synonym of *D.indica*. Both species belong to a distinctive group of Asian species characterized by perulate buds that give rise to shoots bearing flowers or inflorescences proximally and leaves distally, 2–3-carpellate ovaries, and elongate styles. However, *D.nienkui* fundamentally differs from *D.indica* in inflorescence architecture and fruiting pedicel length; these or other characters also distinguish *D.nienkui* from the remaining species in this group. *Drypetesnienkui* therefore deserves recognition as a distinct species endemic to Hainan. An expanded description of the species is provided along with a key to the related species.

## ﻿Introduction

**Figure 1.**
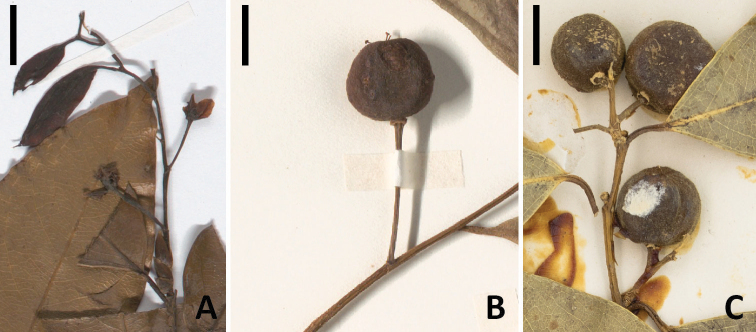
Morphology of the *Drypetesindica* complex **A***D.indica* (detail of L.2207751), branch tip showing perulate bud below an expanding shoot with proximal flowers and distal leaves **B***D.indica* (detail of L.2207743) fruit with elongate styles bearing flabellate stigmas **C***D.nienkui* (detail of NY02684347, holotype) with two cymose infructescences. Scale bars equal 1 cm. Photos **A, B** CC 1.0 Naturalis Biodiversity Center **C** CC 4.0 New York Botanical Garden.

The genus *Drypetes* Vahl (Putranjivaceae Endl., Malpighiales) comprises about 220 species of mostly dioecious trees and shrubs found in the tropics and subtropics worldwide; it is most diverse in Asia, with about 120 species. Thirteen species currently are recognized from China (Qin et al. 2007; Li and Gilbert 2008); of these, nine are reported from Hainan, including the endemic *D.longistipitata* P.T.Li. Among the currently accepted species found in China, there are two, *D.indica* (Müll.Arg.) Pax & K.Hoffm., and *D.longistipitata*, that, along with the Thailand endemic *D.dasycarpa* (Airy Shaw) Phuph. & Chayamarit, belong to a group of taxa that are distinctive among Asian *Drypetes* in having prominent buds enclosed in chartaceous scales (perules; Fig. 1A). When the axillary, and sometimes the terminal, buds develop into shoots, the proximal few nodes usually are leafless and produce flowers or inflorescences, while the more distal nodes produce leaves (Fig. 1A). In addition, the pistils, which are 2–3-carpellate, have more or less elongate styles that bear peltate to flabellate stigmas (Fig. 1B); most other *Drypetes* species in the region have 1-carpellate pistils, sessile stigmas, or both. This group of species, which I will refer to as the *D.indica* complex, ranges from Bhutan and northeastern India eastward to Taiwan and southward to Thailand.

Taxonomic concepts within the *Drypetesindica* complex have varied widely. For example, Pax and Hoffmann (1922), the last to review *Drypetes* worldwide, accepted *D.indica* and four other species that I would place in this complex: *D.griffithii* (Hook.f.) Pax & K.Hoffm., *D.hieranensis* (Hayata) Pax & K.Hoffm., *D.karapinensis* (Hayata) Pax & K.Hoffm., and *D.lancifolia* (Hook.f.) Pax & K.Hoffm.. In contrast, Airy Shaw (1972), in a review of Euphorbiaceae s.l. in Thailand, synonymized the latter four species under *D.indica*. He also treated *D.nienkui* Merr. & Chun, a species described from Hainan, China, as a synonym of *D.indica*. He noted that this treatment made for a highly variable species, but felt that specimens from Thailand and Hainan bridged the morphological and geographic gaps between Indian/Burman material and that from Taiwan. Li (1988) expanded Airy Shaw’s broad concept of *D.indica* to include *D.longipes* X.H.Song, and this view has been followed in treatments of the genus for China (Li 1988, 1994; Li and Gilbert 2008; Fang et al. 2011). However, Taiwanese botanists have continued to treat the material from there as distinct from *D.indica*, recognizing either both *D.hieranensis* and *D.karapinensis* (Hsieh 1977) or, more recently, synonymizing *D.hieranensis* under *D.karapinensis* (Lu 1986; Hsieh et al. 1993; Boufford et al. 2003). Both *D.dasycarpa* and *D.longistipitata* were described after Airy Shaw (1972) expanded the concept of *D.indica* and have consistently been accepted as distinct taxa, although Airy Shaw (1977) initially described *D.dasycarpa* as a variety of *D.indica*.

*Drypetesnienkui* was described from a single fruiting specimen (Merrill and Chun 1935). Because an isotype from this gathering is the only specimen of *D.nienkui* at K (pers. obs. and GBIF 2022) and I have seen no other specimens of this species annotated by Airy Shaw, it appears that he (Airy Shaw 1972) only saw this specimen before synonymizing *D.nienkui* under *D.indica*. Having had the opportunity to examine more specimens, including type material, of *D.nienkui* and the other species in the *D.indica* complex from throughout eastern Asia, I believe the broad treatment of *D.indica* pioneered by Airy Shaw (1972) is too inclusive. *Drypetesnienkui*, in particular, is quite distinct, although further species may also deserve recognition. Here I review morphological characters that distinguish *D.nienkui* from *D.indica* and *D.dasycarpa*; I also provide an expanded description of *D.nienkui* and a key to species I currently accept in the *D.indica* complex.

## ﻿Materials and methods

Morphological characters were examined on specimens held by A, BM, G, IBSC, K, KUN, L, NY, P, and US (herbarium acronyms follow Thiers [continuously updated]), and online images of specimens from AU, CAS, E, HITBC, IBK, MW, NAS, PE, SN, TAI, TI, and TNM available at JSTOR Global Plants (https://plants.jstor.org/), the Chinese Virtual Herbarium (http://www.cvh.ac.cn/), the Global Biodiversity Information Facility (https://www.gbif.org/), and individual herbarium websites. Measurements were made using both actual specimens and specimen images containing scale bars.

## ﻿Results and discussion

Material that Airy Shaw (1972) and others included within *Drypetesindica* exhibits two different inflorescence variants. Specimens from Hainan that have been called *D.indica* or *D.nienkui* have staminate and pistillate flowers borne in cymose inflorescences and pistillate pedicels that are no longer than 12 mm in fruit (Fig. 1C), whereas all the remaining specimens of *D.indica* in the broad sense have fasciculate staminate flowers and solitary pistillate flowers, the pedicels of which become at least 16 mm long in fruit (Fig. 1B); the same is true of *D.longistipitata*. Merrill and Chun (1935) described the infructescences of *D.nienkui* as “depauperate-racemose” (they had only fruiting material), although they actually are cymose. *Drypetesdasycarpa* also has cymose inflorescences and relatively short pistillate pedicels, as was reported by Phuphathanaphong and Chayamarit (2000, 2005). These inflorescence characters were overlooked by Airy Shaw (1972, 1977), perhaps because the specimens of *D.dasycarpa* and *D.nienkui* that he studied at Kew have either only detached fruits or infructescences bearing only a single mature fruit (pers. obs.).

Within the cymose inflorescence group, the specimens sort neatly into two groups based on differences in pubescence, perule size, leaf blade size, staminate pedicel length, and stamen number. The groups correspond to plants from northern and western Thailand (*Drypetesdasycarpa*) and those from Hainan (*D.nienkui*). Within the group with fasciculate staminate flowers and solitary pistillate flowers, it appears that *D.indica* and *D.longistipitata* are distinct, based on differences in leaf blade secondary vein number and prominence, pistillate pedicel length, style length, and drupe size. Specimens of the *D.indica* complex from Taiwan, which have been treated as *D.karapinensis* (of which *D.hieranensis* should be treated as a synonym), differ somewhat from mainland Asian *D.indica* in pubescence, leaf vein number, stamen number, and fruiting pedicel length, but the differences are subtle and overlapping; resolving their taxonomic status is beyond the scope of this paper, and here I treat them both within a broadly defined *D.indica*. Differences among species in the *D.indica* complex are summarized in the key below.

### ﻿Taxonomic treatment

#### ﻿ Drypetes nienkui

Taxon classificationPlantaeMalpighialesPutranjivaceae

Merr. & Chun, Sunyatsenia 2: 258, fig. 29C, D. 1935.

C04C6BF6-0676-530C-B8A3-0B71BA387897

##### Type.

China. Hainan: Fan Ya, Ng Chi Ling, elev. 1200 m, 8 Nov 1932, *N. K. Chun & C. L. Tso 44246* (holotype: NY02684347!; isotypes A00055929!, AU042776! (image seen), IBSC0004245!, IBSC0004246!, IBSC0004247!, K000854225!, NAS00417232! (image seen), PE00022638! (image seen), US01269000!).

##### Description.

Trees to 15(–20) m, to 30 dbh, dioecious; branches and branchlets glabrous. **Buds** perulate; perules ovate, 1.5–2 × 1.6–2 mm, apex obtuse to rounded, surfaces glabrous, margins ciliolate. **Leaves**: stipules caducous, not seen; petiole 8–13 mm long, 1.3–1.7 mm diam., canaliculate, glabrous; blade oblong-ovate to oblong-lanceolate, 8–14 × 3–6 cm, chartaceous-coriaceous, base ± asymmetric, broadly cuneate to broadly acute, margins entire, apex acute to gradually short-acuminate, tip obtuse, surfaces glabrous, abaxial light green, somewhat shiny, adaxial olive, shiny; midvein rounded abaxially and adaxially, 2º veins 6–9 per side, slender but not obscure, 3º reticulate, higher order not well differentiated, reticulate, all raised adaxially, areoles 0.6–1 mm diam., freely ending veinlets fairly common. **Staminate inflorescences** cymose, to 2 cm long, 8–10-flowered; peduncle 0.5–0.8 mm diam., glabrous; bracteoles triangular, 0.8–1 mm long, surfaces glabrous, margins ciliate. **Pistillate inflorescences** cymose, 1 cm long (fruiting), 4–7-flowered; peduncle 1.5–1.7 mm diam. (fruiting), glabrous; bracteoles not seen. **Staminate flowers**: pedicels 2–5 mm long, 0.3 mm diam., puberulent; sepals 4, ovate, 1.7–2 × 1.2–1.5 mm, apex rounded, surfaces glabrous, margins ciliate; disc shallowly lobed, glabrous; stamens 8, inserted between disc lobes, filaments 1.5 mm long, terete, glabrous, anthers +/- globose, 0.5 mm diam., glabrous. **Pistillate flowers** not seen; fruiting pedicels 6–12 mm long, 1.4–1.7 mm diam., puberulent, glabrescent, hairs erect, to 0.1 mm long; ovaries densely hirsute when young. **Drupes** purplish brown, globose or depressed globose, sometimes slightly lobed, 15–18 mm diam., 2–3-locular, surface sparsely hirsute, trichomes whitish, 0.1–0.2 mm long, erect; styles 2–3, 1.5–2 mm long; stigmas +/- flabellate, 0.5 × 0.7 mm; exocarp and mesocarp not differentiated, 0.5 mm thick, leathery or crustaceous, endocarp 0.1 mm thick, cartilaginous. **Seeds** 2–3.

##### Phenology.

*Drypetesnienkui* flowers August-November and fruits November–January.

##### Distribution and habitat.

*Drypetesnienkui* is widespread in the southern half of Hainan, China (see the map for *D.indica* in Fang et al. [2011], as to Hainan only), occurring in forests at 950–2100 m elevation.

##### Preliminary conservation status.

I have been able to locate only 13 gatherings of *Drypetesnienkui*. With the exception of two collections made in 2014 and 2017, all the collections date from between 1932 and 1954, but no data are available regarding population sizes or trends. Applying the criteria of IUCN (2022), *D.nienkui* should receive a preliminary conservation assessment of Data Deficient, although a more complete assessment might show it qualifies as a species of elevated conservation concern.

### ﻿Key to species in the *Drypetesindica* complex

**Table:** 

1	Pistillate and staminate inflorescences cymose; fruiting pedicels 5–12 mm long	**2**
–	Pistillate flowers solitary, staminate inflorescences fascicles (unknown in *D.longistipitata*); fruiting pedicels 16–40 mm long	**3**
2	Young stems and petioles hairy; perules 5–7 mm long, densely hairy; leaf blades 10–18 cm long; staminate flowers: pedicels 5–10 mm long, disc hairy, stamens 8–12	***Drypetesdasycarpa***
–	Young stems and petioles glabrous; perules 1.5–2 mm long, glabrous except for ciliate margins; leaf blades 8–14 cm long; staminate flowers: pedicels 3–5 mm long, disc glabrous, stamens 8	***Drypetesnienkui***
3	Leaf blade secondary veins 5–8 pairs, prominent; fruiting pedicels 20–40 mm long; styles 1.5–3 mm long; drupes 12–16 mm diam	***Drypetesindicas.l.* (including *D.karapinensis*)**
–	Leaf blade secondary veins 4–5(–6) pairs, delicate; fruiting pedicels 16–20 mm long; styles 1 mm long; drupes 10–12 mm diam	***Drypeteslongistipitata***

## Supplementary Material

XML Treatment for Drypetes nienkui

## ﻿Appendix 1

**Specimens examined for comparative morphological studies of the *Drypetesindica* complex.** Listed are country, primary collector name and collection number, date of collection, herbarium, and, when available, barcodes (enclosed in square brackets when they do not include the herbarium acronym) or accession numbers (identified by “acc. #”). Specimens that I observed only through online images are marked with an asterisk (*).

***Drypetesnienkui***: China. *N. K. Chun 44246*, 8-Nov-1932, A00055929, AU042776*, IBSC0004245, IBSC0004246, IBSC0004247, K000854225, NAS00417232*, NY02684347, PE00022638*, US01269000; *Chun W. Y. 420*, 26-Nov-1936, IBSC0311573; *Hainan East Road team 610*, 9-Nov-1954, IBK00165777*, IBSC0311570; *Hainan East Road team 960*, 19-Dec-1954, IBK00165780*, IBK00165784*, IBSC0311574; *Hainan hanging mountain team 2988*, 19-Dec-1954, IBK00165775*, IBSC0311575; *How F. C. 73525*, 29-Aug-1935, AU012778*, IBK00165774*, IBK00165781*, IBSC0311578, NAS00412112*; *How F. C. 73673*, 15-Sep-1935, IBK00165782*, IBSC0311579; *Liu C. 17CX15977*, 10-Sep-2017, KUN1445422*; *Wang C. 35261*, 9-Dec-1933, IBK00165776*, IBSC0311572, NAS00412111*, NY; *Wang C. 36145*, 6-Jan-1934, IBK00165779*, IBSC0311576; *Wang C. 36238*, 9-Jan-1934, IBSC0311577; *Wang C. 36622*, 13-Jan-1934, IBK00165778*, IBSC0311571, SN007954*, NY; *Zhang T. 14CS8905*, 19-Aug-2014, KUN1371389.

***Drypetesdasycarpa***: Thailand. *N. Fukuoka T-62161*, 14-Jan-1994, L.2207742; *H. B. G. Garrett* 593, 6-Nov-1930, BM, P05470830*; *A. F. G. Kerr 4919*, 26-Feb-1921, P05563357; *A. F. G. Kerr 5047*, 8-Mar-1921, BM; *H. P. Nooteboom 807*, 21-Jan-1969, K000854258, L.2207753; *T. Yahara T-50009*, 10-Dec-1984, L.2207754.

***Drypetesindica**s.l.* (including *D.karpapinensis*)**: Bhutan. *A. J. C. Grierson 1694*, 6-Jun-1979, E00310566*. China. *Li Y.-H. 12533*, 28-Jun-1974, HITBC Acc. # 014370*, 081461*. India. *W. Griffith 4738*, K000246683; *J. D. Hooker s.n.*, E00310592*, G00318470, G00318473, K000246684, K000246685, K000246686, K000246687, L.2207755, L.2208087, L.2208088, P05470767, P05470831, P05470832, P05470835, P05470836, P05470837, P05470838. Taiwan. *C.-H. Chen 505*, 1-Apr-1994, TNM [S14022*, S34201*]; *S. T. Chiu 1657*, 31-Mar-1993, TNM [S10365*, S10367*]; *T. I. Chuang 3117*, 10-Jan-1960, TAI Acc. # 067424*, Acc. # 067425*; *S.-W. Chung 5062*, 26-Feb-2002, TNM [S79934*]; *S.-W. Chung 5072*, 26-Feb-2002, TNM [S79933*]; *S.-W. Chung 5073*, 26-Feb-2002, TNM [S79935*]; *Hayata 45*, 23-Apr-1914, A00055901, IBSC0311581, TAI Acc. #01604*, Acc. # 01605*; *H.-Y. Chen 151*, 8-Mar-1998, TNM [S77423*]; *H.-Y. Chen 163*, 8-Mar-1998, TAI Acc. # 251490*; *G.-P. Hsiah 1383*, 1-May-2004, TNM [S108917*]; *Y.-Y. Huang, 327*, 29-Apr-2021, CAS669031*; *V.-P. Leu s.n.*, 6-Apr-1939, TNM [S3042*]; *S.-P. Li 435*, 2-Mar-1995, IBSC0311582, TNM [S172508]; *S.-Y. Lu 256*, 2-Apr-1993, TAI Acc. # 225015*; *S.-Y. Lu 18279*, 6-Feb-1986, L.2207711, MW0756562*, MW0756562*; *S.-Y. Lu 18606*, 11-Mar-1986, L.3799848; *S.-Y. Lu 18613*, 13-Dec-1982, L.2207709; *Ou 9452*, 31-May-1980, TAI Acc. #178887, Acc. # 178895; *S. Sasaki s.n.*, Feb 1912, TI Acc. #01603*; *C. M. Wang 2126*, 25-Feb-1996, CAS669034*; *J.-C. Wang 8177*, 24-Apr-1993, TAI Acc. # 255401; *L. J. Wang s.n.*, 19-Mar-1966, TAI Acc. # 067420*, Acc. # 067421*; *S. Z. Yang 10703*, 10-Apr-1988, IBSC0311565; *S. Z. Yang 10761*, 19-Mar-1989, IBSC0311564; *T. Y. A Yang 23063*, 27-Feb-1997, TNM [S31964*]. Thailand, *A. F. G. Kerr 12049*, 12-Feb-1927, BM, L.2207741, P05470829; *A. F. G. Kerr 16780*, 21-Jan-1929, BM, L.2207744; *A. F. G. Kerr 17181*, 22-Feb-1929, BM, P.05563358; *A. F. G. Kerr 17184*, 22-Feb-1929, BM, P05563356; *A. F. G. Kerr 17991*, 7-Jan-1930, L.2207749; *A. F. G. Kerr 18004*, 7-Jan-1930, BM, L.2207748; *A. F. G. Kerr 18442*, 8-Mar-1930, L.2207747; *A. F. G. Kerr 18448*, 5-Mar-1930, BM; *A. F. G. Kerr 18448A*, 9-Mar-1930, BM, L.2207745, P05563359.

***Drypeteslongistipitata***: China. *How F. C. 70367*, 17-Mar-1933, IBK00165773*; *Wei C. F. 122372*, 24-Apr-1959, IBSC0311561; *Xing Z. W. 6196*, 16-May-1995, IBSC0311562, IBSC0311563.

## References

[B1] Airy Shaw HK (1972) The Euphorbiaceae of Siam. Kew Bulletin 26(2): 191–364. 10.2307/4117717

[B2] Airy Shaw HK (1977) Additions and corrections to the Euphorbiaceae of Siam. Kew Bulletin 32(1): 69–83. 10.2307/4117261

[B3] Boufford DE Ohashi H Huang TC Hsieh CF Tsai JL Yang KC Peng C-I Kuoh CS Hsiao A (2003) A checklist of the vascular plants of Taiwan. In: Huang T-C et al. (Eds) Flora of Taiwan, ed. 2, vol. 6. Editorial Committee of the Flora of Taiwan, Taipei, 15–139. https://tai2.ntu.edu.tw/ebooks/FlTaiwan2nd/6

[B4] Fang J Wang Z Tang Z (2011) Atlas of Woody Plants in China: Distribution and Climate. Springer, Berlin, 2000 pp. 10.1007/978-3-642-15017-3

[B5] GBIF [continuously updated] (2022) GBIF Occurrence Download. 10.15468/dl.cmeyk2 [accessed 26 August 2022]

[B6] Hsieh CF (1977) Euphorbiaceae. In: Li HL Liu TS Huang TC Koyama T DeVol CE (Eds) Flora of Taiwan, ed.1, vol. 3. Epoch Publishing Co., Taipei, 436–500. https://tai2.ntu.edu.tw/ebooks/FlTaiwan/3

[B7] Hsieh CF Chaw SM Wang JC (1993) Euphorbiaceae. In: Huang T-C et al. (Eds) Flora of Taiwan, ed. 2, vol. 3. Editorial Committee of the Flora of Taiwan, Taipei, 414–504. https://tai2.ntu.edu.tw/ebooks/FlTaiwan2nd/3

[B8] IUCN (2022) Guidelines for Using the IUCN Red List Categories and Criteria. Version 15. Prepared by the Standards and Petitions Subcommittee, 114 pp. https://nc.iucnredlist.org/redlist/content/attachment_files/RedListGuidelines.pdf

[B9] Li P-T (1988) Materials for Chinese Phyllanthoideae. Zhiwu Fenlei Xuebao 26: 58–65. https://www.jse.ac.cn/EN/Y1988/V26/I1/58

[B10] Li P-T (1994) Angiospermae-Dicotyledoneae-Euphorbiaceae-Phyllanthoideae. In: Li P-T (Ed.) Flora Republicae Popularis Sinicae, vol.44(1). Science Press, Beijing, 1–207

[B11] Li P-T Gilbert MG (2008) *Drypetes* (Euphorbiaceae). In: Wu Z Raven PH (Eds) Flora of China, vol.11. Science Press and Missouri Botanical Garden Press, Beijing and St. Louis, 218–221.

[B12] Lu SY (1986) The genus *Drypetes* (Euphorbiaceae) in Taiwan. Quarterly Journal of Chinese Forestry 19: 99–105.

[B13] Merrill ED Chun WY (1935) Additions to our knowledge of the Hainan flora, II. Sunyatsenia 2: 203–332.

[B14] Pax F Hoffmann K (1922) Euphorbiaceae-Phyllanthoideae-Phyllantheae. In: Engler HGA (Ed.) Das Pflanzenreich, IV, 147.XV (Heft 81), Verlag der Wilhelm Engelmann, Leipzig, 1–349.

[B15] Phuphathanaphong L Chayamarit K (2000) *Drypetesdasycarpa* (Airy Shaw) Phuph. & Chayamarit stat. nov. (Euphorbiaceae). Thai Forestry Bulletin (Botany) 28: 160–162.

[B16] Phuphathanaphong L Chayamarit K (2005) *Drypetes*. In: Chayamarit K van Welzen PC (Eds) Flora of Thailand, vol.8(1), Euphorbiaceae (Genera A-F). The Forest Herbarium, National Park, Wildlife and Plant Conservation Department, Bangkok, Thailand, 231–253.

[B17] Qin X-S Chen H-F Xing FW (2007) A new species of *Drypetes* (Putranjivaceae) from China. Nordic Journal of Botany 25(1–2): 38–40. 10.1111/j.0107-055X.2007.00139_3.x

[B18] Thiers B ([continuously updated] 2022) Index Herbariorum: A global directory of public herbaria and associated staff. http://sycamore.nybg.org/science/ih/ [accessed 10 Jun 2022]

